# Engineered Interspecies Amino Acid Cross-Feeding Increases Population Evenness in a Synthetic Bacterial Consortium

**DOI:** 10.1128/mSystems.00352-19

**Published:** 2019-08-13

**Authors:** Marika Ziesack, Travis Gibson, John K. W. Oliver, Andrew M. Shumaker, Bryan B. Hsu, David T. Riglar, Tobias W. Giessen, Nicholas V. DiBenedetto, Lynn Bry, Jeffrey C. Way, Pamela A. Silver, Georg K. Gerber

**Affiliations:** a Wyss Institute for Biologically Inspired Engineering, Harvard Medical School, Boston, Massachusetts, USA; b Department of Systems Biology, Harvard Medical School, Boston, Massachusetts, USA; c Massachusetts Host-Microbiome Center, Brigham and Women’s Hospital, Harvard Medical School, Boston, Massachusetts, USA; Rensselaer Polytechnic Institute

**Keywords:** metabolite cross-feeding, microbial consortia, synthetic biology

## Abstract

In nature, microbes interact antagonistically, neutrally, or beneficially. To shed light on the effects of positive interactions in microbial consortia, we introduced metabolic dependencies and metabolite overproduction into four bacterial species. While antagonistic interactions govern the wild-type consortium behavior, the genetic modifications alleviated antagonistic interactions and resulted in beneficial interactions. Engineered cross-feeding increased population evenness, a component of ecological diversity, in different environments, including in a more complex gnotobiotic mouse gut environment. Our findings suggest that metabolite cross-feeding could be used as a tool for intentionally shaping microbial consortia in complex environments.

**IMPORTANCE** Microbial communities are ubiquitous in nature. Bacterial consortia live in and on our body and in our environment, and more recently, biotechnology is applying microbial consortia for bioproduction. As part of our body, bacterial consortia influence us in health and disease. Microbial consortium function is determined by its composition, which in turn is driven by the interactions between species. Further understanding of microbial interactions will help us in deciphering how consortia function in complex environments and may enable us to modify microbial consortia for health and environmental benefits.

**Author Video**: An author video summary of this article is available.

## Abstract (precis)

Microbial communities are ubiquitous in nature. Bacterial consortia live in and on our body and in our environment, and more recently, biotechnology is applying microbial consortia for bioproduction. As part of our body, bacterial consortia influence us in health and disease. Microbial consortium function is determined by its composition, which in turn is driven by the interactions between species. Further understanding of microbial interactions will help us in deciphering how consortia function in complex environments and may enable us to modify microbial consortia for health and environmental benefits.

## INTRODUCTION

In nature, microbes occur as conglomerates of various species with diverse sets of genomes and metabolic capabilities (1). Composition of these consortia arises from networks of ecological interactions and determines function and performance in various environments. Ecological interactions can be antagonistic (e.g., competition for nutrients), neutral (no interaction), or beneficial (e.g., resource sharing) (2). Studies suggest that both antagonistic and beneficial interactions drive microbial consortium composition (3). According to classic evolutionary theory, competitive interactions favoring survival of one species over another should be prevalent, and a body of experimental research supports that hypothesis (4–6). Recent studies of the microbiota also highlight the importance of beneficial interactions, particularly those that provide “public goods” (7, 8). “Public goods” are metabolites that are released into the environment and benefit not only the producer but also neighboring cells.

A meta-analysis of 77 studies revealed that cross-feeding of metabolites between naturally occurring bacteria is common (7). Metabolite cross-feeding may be explained by comparative advantages of metabolic capabilities in some species over others and removal of biochemical conflicts in biosynthetic pathways within cells (9, 10). The evolution of such mutually beneficial interactions may occur as follows. Metabolic leakiness in strain A can serve as the first step toward metabolic dependencies as it results in products leaking into the environment where neighboring strain B can take them up (11, 12). Strain B may lose the ability to make the metabolites itself, resulting in auxotrophy. Indeed, microbial auxotrophies are common in natural communities. In a study of 949 eubacterial genomes, 76% were found to lack at least one of 25 different metabolic biosynthesis pathways (13).

There is increasing recognition that maintaining species diversity—a measure of richness and evenness within a population—in microbiomes is important to ecosystem health (14). A study that analyzed composition of a syntrophic consortium of methanotrophs found that amino acid cross-feeding may help with retaining species diversity, which leads to higher robustness with regard to limiting loss or dominance of species in the consortium (15). Despite the fact that bacteria competed for similar nutritional niches, the consortium composition remained stable. Thus, metabolite cross-feeding could serve as a mechanism to preserve species diversity by limiting the ability of some consortium members to dominate and eliminate other species.

Amino acid cross-feeding is also an attractive means to introduce cooperation into synthetic microbial consortia. Numerous studies have engineered pairwise amino acid cross-feeding in Escherichia coli with native amino acid production levels and generated quantitative models to describe their behavior (16–21). Most prior synthetic biology studies that have engineered cooperativity in bacterial communities have used a single species (21–24). However, natural microbial ecosystems contain a diversity of interacting species.

In advancing synthetic biology to real applications in complex environments, it will be useful to expand engineering capabilities to diverse, multispecies consortia. Importantly, bacteria from naturally occurring ecosystems are likely to have preexisting interactions, which are often competitive. In a given bacterial consortium that is dominated by antagonistic interactions, synthetically introduced positive interactions need to overcome these antagonistic interactions in order to cause a net beneficial effect. Here, we engineer metabolite cross-feeding in a synthetic consortium of four different bacterial species and demonstrate net beneficial behavior. We further observe increased population evenness—a component of species diversity within a population—in different environments *in vitro* and *in vivo*.

## RESULTS

### Engineering of metabolic dependency and overproduction between four species.

**FIG 1:**
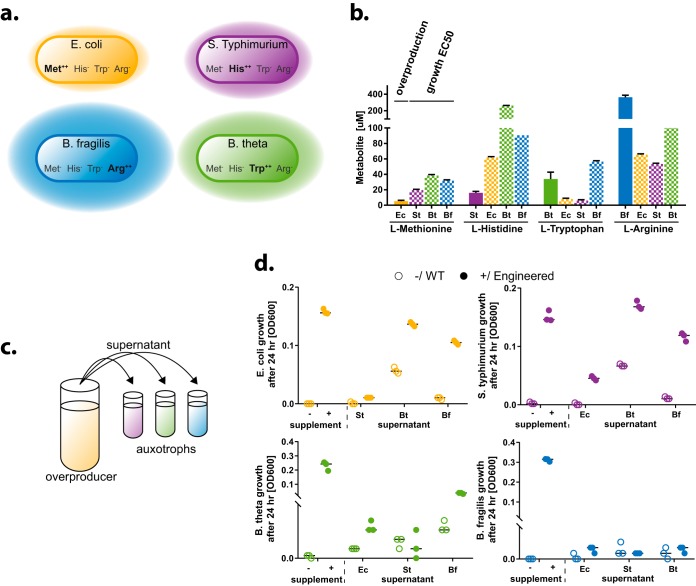
Engineered metabolite overproduction and growth requirements. (a) Consortium design. Each strain is auxotrophic for three amino acids and overproduces one. (b) Quantification of overproduction and growth requirement (EC_50_). Overproduction in each engineered strain was measured via LC-MS with an appropriate amino acid standard. Metabolite growth requirement EC_50_ was measured in medium with full supplementation of two amino acids and various amounts of one other. (c) Cross-feeding capabilities of each strain were assessed by testing for rescue of auxotroph growth in supernatants obtained from overproducers. (d) Growth assay of auxotrophic strains. Auxotroph strains were grown in medium without and with addition of all cross-fed amino acids (left side of graphs, −/+), which served as a reference point. Each strain was also grown in fresh medium without supplementation and supernatant of engineered overproducers or WT counterpart at a 1:1 ratio. Shown are three biological replicates with medians indicated as horizontal lines.

For our consortium, we chose four bacterial species that naturally reside in the mammalian gut, a complex environment of intense interest from both the basic scientific and biomedical perspectives (Fig. 1a). The organisms were chosen in part for their propensity to colonize in the colon, which we hypothesized could allow for sufficient cross-feeding opportunities. A second crucial criterion for the selected organisms was the availability of tools for efficient genetic manipulation; to date, a very small subset of commensal gut microbes are genetically tenable, and thus, a wider range of organisms was not feasible. Nonetheless, these organisms represent gut residents with different spatial niches and carbohydrate utilization preferences: E. coli and Salmonella enterica serovar Typhimurium tend to utilize simpler carbohydrates as found in the gut lumen (25, 26), whereas *Bacteroides* spp. tend to be mucosally associated and utilize more complex carbohydrates (27).

Escherichia coli NGF-1 was originally isolated from BALB/c mice, has been shown to stably colonize the mouse gut, and can be engineered with standard genetic tools (28–30). Salmonella enterica serovar Typhimurium LT2 (*S.* Typhimurium), an attenuated gut pathogen, was rendered further harmless by removing the pathogenicity islands SPI1 and SPI2 and thus causes no disease when administered to mice (31). The two *Bacteroides* species, Bacteroides thetaiotaomicron VPI-5482 and Bacteroides fragilis 638R, are human commensals that can achieve high abundance in the mammalian gut and are also genetically tractable.

We engineered each of the constituent species to depend on three of the four metabolites l-methionine, l-histidine, l-tryptophan, and l-arginine (here referred to as Met, His, Trp, and Arg, respectively) and to overproduce the remaining amino acid (Fig. 1a). As we noted, amino acid cross-feeding has been described as a prominent natural mechanism (7) and has previously been used extensively in single-species engineering efforts. Our rationale for choosing these specific amino acids for cross-feeding was in part based on the feasibility of engineering overproduction and auxotrophies in the selected species based on prior work. Additionally, we considered the amino acid composition of proteins across a variety of bacterial taxa, which indicated lower use of Met, His, and Trp and higher use of Arg (32). We therefore hypothesized that these particular amino acids would offer a range of nutrient requirements for testing and evaluating consortium behavior.

The auxotrophies introduced for each strain were as follows: E. coli, His, Trp, and Arg; *S.* Typhimurium, Met, Trp, and Arg; B. thetaiotaomicron, Met, His, and Arg; B. fragilis, Met, His, and Trp. E. coli and *S.* Typhimurium were engineered by sequential phage transduction from three single auxotroph strains. E. coli was transduced with genome fragments from BW25113 that contained insertions in *argA*, *trpC*, and *hisA* (see Materials and Methods), and *S*. Typhimurium was transduced with genome fragments of the same parent strain with insertions in *argA*, *trpC*, and *metA*. *Bacteroides* triple knockout generation utilized the pExchange-tdk vector to precisely delete *metA*, *hisG*, and *argF* in B. thetaiotaomicron and *metA*, *hisG*, and *trpC* in B. fragilis. We introduced the ability to overproduce one amino acid in each strain by using antimetabolite selection methods (E. coli, Met; *S.* Typhimurium, His; B. thetaiotaomicron, Trp; B. fragilis, Arg) and identified putatively causal mutations by sequencing and single nucleotide polymorphism (SNP) analysis (Table 1 and Materials and Methods).

**TABLE 1:** Engineered strains, engineered genotypes, and a subset of identified SNPs previously implicated as causal mutations

Species	Strain	Auxotroph genotype	Other genotype	Overproduction mutation(s)
*E. coli*	NGF	Δ*argA* Δ*trpC* Δ*hisA*	Δ*thiE*	*metA* (I296S)^*a*^
*S.* Typhimurium	LT2	Δ*argA* Δ*trpC* Δ*metA*	Δ*thiE* ΔSPI1 ΔSPI2	*hisG* (E271K)^*b*^
*B. thetaiotaomicron*	VPI 5482	Δ*metA* Δ*hisG* Δ*argF*	Δ*thiSEG* Δ*tdk*	BT_0532 (A306V; N63D)^*c*^
*B. fragilis*	368R	Δ*metA* Δ*hisG* Δ*trpC*	Δ*thiSEG* Δ*tdk*	BF638R_0532 (L26R)^*d*^

a Prevents feedback inhibition (45).

b Decouples from histidine feedback inhibition (46).

c TrpE, removes feedback inhibition (47).

d Arginine repressor, nonfunctional (48).

To assess the auxotrophic strains’ requirements for the metabolite, we measured growth on various concentrations of each metabolite in the presence of nonlimiting concentrations of all the other metabolites (see Fig. S1 in the supplemental material). We found that each strain has distinct requirements for the amino acids for which it was auxotrophic. In general, the *Bacteroides* spp. have higher amino acid requirements than E. coli and *S*. Typhimurium, which could in part be due to differences in their capability to transport/take up amino acids. We note also that the observed amino acid requirements correlate with differing amino acid compositions in proteins of these species (33).

10.1128/mSystems.00352-19.1 FIG S1 Growth response. Each auxotroph was grown in medium supplemented with various concentrations of one amino acid and saturating concentration of the two others. Depicted is the average from three biological replicates; error bars indicate standard deviations. A sigmoidal curve was fitted using GraphPad Prism 8. Download FIG S1, EPS file, 2.1 MB. Copyright © 2019 Ziesack et al. 2019 Ziesack et al. This content is distributed under the terms of the Creative Commons Attribution 4.0 International license.

Overproduction of metabolites was measured in comparison to a defined amino acid standard using liquid chromatography-mass spectrometry (LC-MS) (Fig. 1b, solid bars, and Fig. S6). In order to compare overproduction with each species’ amino acid requirements, we fitted a sigmoidal curve to the growth response data and calculated the 50% effective concentration (EC_50_) requirement (Fig. 1b, shaded bars, and Fig. S1). In this context, the EC_50_ requirement describes the concentration of supplemented metabolite that allows for half-maximum growth of the respective strain. These data on amino acid requirements and overproduction levels provide information about the expected relative strengths of the engineered beneficial interactions.

Engineered B. fragilis overproduced Arg at 362 μM—a concentration exceeding EC_50_ requirements of any of the other strains. Corresponding supplementation allows for more than half-maximum growth of either one of the strains. B. thetaiotaomicron overproduced Trp at 34 μM, which exceeded the EC_50_ requirements of E. coli and *S*. Typhimurium but not B. fragilis. Overproduction from E. coli (Met at 5.2 μM) and *S*. Typhimurium (His at 16 μM) was lower than any of the EC_50_ requirement values. EC_50_ requirement values exceeded E. coli Met overproduction by 4-, 7-, and 6-fold and *S.* Typhimurium His overproduction by 12-, 51-, and 17-fold. Overall, these findings suggest relatively strong cross-feeding from *Bacteroides* spp., moderate cross-feeding from E. coli, and the weakest cross-feeding from *S*. Typhimurium to other strains. Note that wild-type (WT) E. coli and *S*. Typhimurium do not produce detectable levels of the respective amino acids in supernatant, whereas WT B. thetaiotaomicron produces 5.7 ± 0.8 μM Trp and WT B. fragilis produces 1.7 ± 1.2 μM Arg (Fig. S6).

To provide further insight into the ability of each overproducer strain to support the auxotrophic strains, we performed assays in which each auxotrophic strain was grown in the presence of supernatant from the overproducer strain or its WT counterpart (Fig. 1c). Overall, our overproduction and requirement assay described above predicted growth in the supernatant assay. The majority of WT strain supernatants supported no to minimal growth of the auxotrophs, whereas the overproducer supernatants variably supported growth depending on the match between overproduction and metabolite requirements of the respective pair of strains (Fig. 1d). Exceptions to this observation were WT B. thetaiotaomicron supernatant, which supported some growth of E. coli and *S.* Typhimurium, and WT B. fragilis supernatant, which supported some growth of B. thetaiotaomicron. This is consistent with our finding that the WT strains of these organisms produced a small amount of the respective amino acid (Fig. S6). Growth of E. coli was rescued by supernatant from overproducing B. thetaiotaomicron and B. fragilis but not *S.* Typhimurium. *S.* Typhimurium growth was rescued by all overproducer supernatants. B. thetaiotaomicron growth was partially rescued by E. coli and B. fragilis but not *S.* Typhimurium overproducer supernatant. B. fragilis growth was not rescued by any of the strains’ overproducer supernatants but is rescued in coculture (see below).

### Engineered cross-feeding introduces beneficial interactions in the consortium.

To characterize the behavior and interaction structure of the consortium as a whole, we performed a set of experiments in which we cultured all members together while varying the starting inoculum of one member at a time. By measuring the growth trajectories of all strains simultaneously, we can analyze how fluctuations in the growth of one member of the consortium influences changes in the growth of other members over time and thus infer the interactions that are occurring. We performed these experiments under five conditions for both WT and engineered consortia.

**FIG 2:**
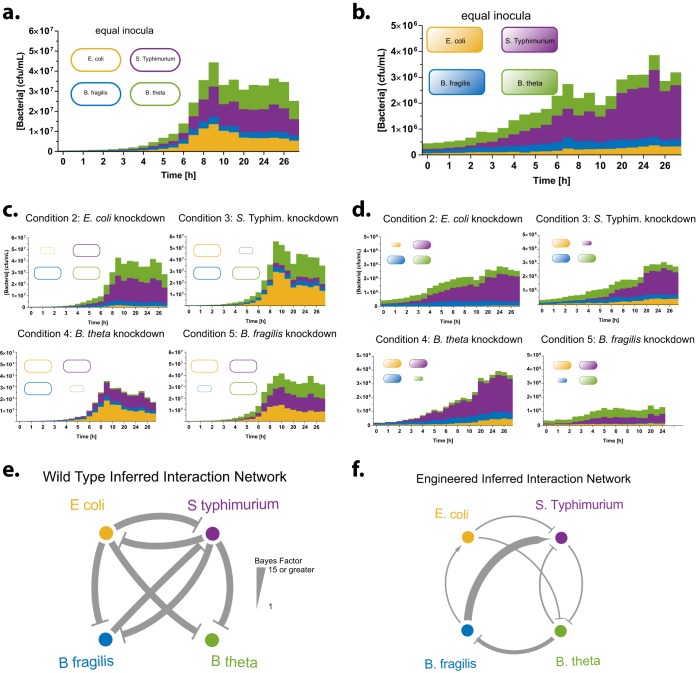
Engineered consortium exhibits added beneficial interactions and reduced antagonistic interactions. (a and b) Growth trajectories in anaerobic batch cocultures for WT (a) and engineered (b) consortia. All strains were inoculated at equal ratios and grown anaerobically at 37°C for 27 h in M9 minimal medium with specific modifications as described in Materials and Methods, without supplementation of any of the cross-fed amino acids, and with 0.5% starch and 0.5% glucose as carbon sources. The engineered consortium grows to an order-of-magnitude-lower density than the WT counterpart. (c and d) Growth trajectories of consortia with each strain inoculum reduced by 10-fold from WT (c) and engineered (d) consortia. These data combined were used to infer a network interaction using the MDSINE algorithm. (e and f) Inferred interaction networks for WT (e) and engineered (f) consortia. WT consortia show mainly negative interactions with large Bayes factors. Engineered consortia’s negative interactions have reduced Bayes factors, and positive interactions with strong Bayes factors occurred between B. fragilis and E. coli and *S.* Typhimurium.

Under condition 1, which served as the baseline, we inoculated all consortium members at equal ratios (Fig. 2a and b), and under conditions 2 to 5, we reduced the starting inoculum of each of the four species in turn by a factor of 10 (Fig. 2c and d). We grew the consortia anaerobically at 37°C for 27 h in M9 minimal medium with specific modifications as described in Materials and Methods, without supplementation of any of the cross-fed amino acids, and with 0.5% starch and 0.5% glucose as carbon sources. We assessed growth of each strain in coculture over time via strain-specific quantitative PCR (qPCR).

We were able to record growth of each strain in WT consortia, indicating that conditions were permissive for each strain (Fig. 2a). We also recorded growth of each strain in the engineered consortia, which indicates that cross-feeding capabilities were sufficient to support growth (Fig. 2b). As expected based on our previous experiment, the engineered consortia did not fully rescue all of the auxotrophies and hence grew to about 10-fold lower overall CFU/ml. In the WT consortium, knockdown of one species persists throughout culturing, whereas in the engineered consortium *S.* Typhimurium and B. thetaiotaomicron are able to recover to baseline ratios (Fig. 2c and d). Two further characteristics of the engineered consortia are notable. Under all five conditions, *S.* Typhimurium appears to dominate the culture, and B. fragilis knockdown causes a decrease in overall consortium growth by 2-fold.

We note that no B. fragilis growth in supernatant was observed, whereas we did see growth in the coculture setting. This difference may be due to several reasons. One possibility is that in the coculture setting B. fragilis may have time to grow to sufficient biomass such that the growth inhibition is blunted, whereas in the supernatant setting the other organisms can accumulate high concentrations of substances that inhibit initial B. fragilis growth. Alternately, other consortium members may be able to actively buffer negative effects. For example, we have observed that *S.* Typhimurium acidifies the medium, which, without appropriate buffering, strongly decreased *Bacteroides* growth (data not shown).

To visualize how lowering the inoculum of each strain (“knockdown”) affected growth of the other strains, we plotted log-fold ratios (logR) comparing the knockdown condition to the equal-inoculum condition (Fig. S2 and S3). Positive logR values for strain A indicate more growth of that strain in the absence of the knockdown strain B, suggesting a net antagonistic effect of strain B on strain A; conversely, a negative logR value suggests a net positive effect. We see either negligible or positive logR values for the WT consortium (Fig. S2), suggesting that it is dominated by antagonistic interactions. In contrast, for the engineered consortium we see negligible or negative logR values (Fig. S3), suggesting that antagonistic interactions have been reduced and positive interactions have been introduced.

10.1128/mSystems.00352-19.2 FIG S2 Log fold ratios (logR) trajectories in WT consortium under all four conditions compared to condition 1 in Fig. 2. Abbreviations: Ec, E. coli; ST, *S.* Typhimurium; BT, B. thetaiotaomicron; BF, B. fragilis. Download FIG S2, EPS file, 1.0 MB. Copyright © 2019 Ziesack et al. 2019 Ziesack et al. This content is distributed under the terms of the Creative Commons Attribution 4.0 International license.

10.1128/mSystems.00352-19.3 FIG S3 Log fold ratios (logR) trajectories in engineered consortium under all four conditions compared to condition 1 in Fig. 2. Abbreviations: Ec, E. coli; ST, *S.* Typhimurium; BT, B. thetaiotaomicron; BF, B. fragilis. Download FIG S3, EPS file, 1.0 MB. Copyright © 2019 Ziesack et al. 2019 Ziesack et al. This content is distributed under the terms of the Creative Commons Attribution 4.0 International license.

To quantitate interactions present in the consortium, taking into account both direct and cascading effects, we used a statistical method based on well-established techniques for inferring microbial interaction networks from time-series data (33–36). Briefly, the method models the rate of change in growth of species A as a function of the concentrations of itself and all other species present in the ecosystem. (See Materials and Methods for further details.) Applying our inference method to the data presented in Fig. 2a to d, we obtained a network of inferred interactions and accompanying estimates of confidence in each edge in the network (Fig. 2e and f and Fig. S4). Consistent with our qualitative visualization, all inferred interactions for the WT consortium were antagonistic (Fig. 2e). In contrast, we see two inferred positive interactions in the engineered consortium network, from B. fragilis to E. coli and *S.* Typhimurium with high inference confidence, which is also consistent with our qualitative visualization (Fig. 2f). Further, although some inferred negative interactions are still present in the engineered consortium network, the confidence in these predictions is much lower than in the WT network, indicating that the model finds greatly reduced evidence for antagonistic interactions in the engineered consortium. Taken together, our findings indicate that introducing metabolic dependencies and overproduction capacities alleviated antagonistic interactions and introduced some beneficial interactions in the consortia.

10.1128/mSystems.00352-19.4 FIG S4 Growth rates, Bayes factors (for microbial interactions), and microbial interaction strengths for unengineered (A) and engineered (B) consortia learned from *in vitro* growth data. The inferred growth rates for the engineered consortia are smaller by about an average factor of 3 for any species. The Bayes factors quantify the evidence for an interaction being present versus being absent (Bayes factor between 3 and 10 = substantial evidence, Bayes factor of >10 = strong evidence). The inference is highly confident in 7 of the 12 unenginereerd consortium interactions. Note that an infinite Bayes factor means that the edge was included in every single step when learning the posterior distribution. For the engineered consortia, two of the 12 interactions exist with at least substantial evidence with 5 of the interactions having very low evidence for the edge being present in the model but still greater than evidence for no interaction (Bayes factor of 1 is the scenario where evidence for and evidence against an interaction being present are equal). The interaction strengths for the engineered consortia are of higher magnitude than for the unengineered consortia. This occurs because the carrying capacity of the unengineeered consortia is about 1.5 orders of magnitude larger than the engineered consortia and microbe-microbe interactions are inversely proportional to the carrying capacity in a gLV model. Download FIG S4, EPS file, 0.7 MB. Copyright © 2019 Ziesack et al. 2019 Ziesack et al. This content is distributed under the terms of the Creative Commons Attribution 4.0 International license.

### Engineered consortia show higher population evenness than their WT counterpart *in vitro*.

**FIG 3:**
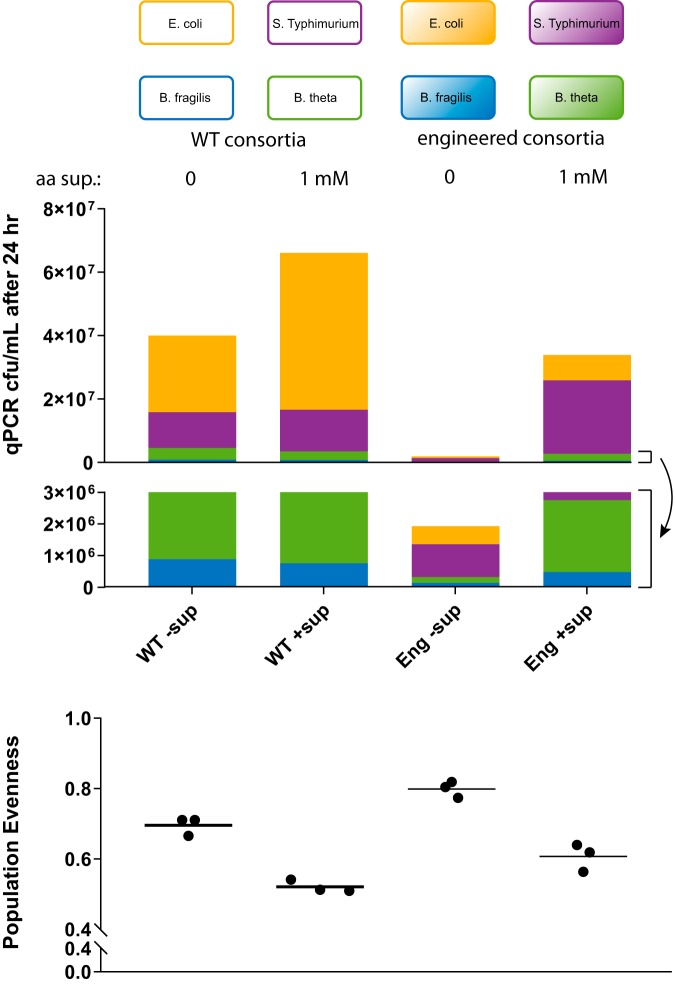
Engineered consortia exhibit increased population evenness *in vitro*. WT and engineered consortia were grown anaerobically in batch coculture for 24 h anaerobically in modified M9 medium as described in Materials and Methods with and without supplementation of 1 mM cross-fed amino acids and 0.2% starch and 0.5% glucose as carbon sources. (Upper panel) Average population composition after 24 h. (Lower panel) Quantified Pielou evenness. The engineered consortium has higher population evenness with or without amino acid supplementation than the WT consortium.

To gain insight into how beneficial interactions affect consortium behavior, we compared consortium compositions in environments with different nutrient levels (Fig. 3). We grew consortia in batch culture for 24 h anaerobically in modified M9 medium as described in Materials and Methods with and without supplementation of 1 mM concentrations of the cross-fed amino acids and 0.2% starch and 0.5% glucose as carbon sources. As described for the previous trajectory experiment, the engineered consortia were able to grow without amino acid supplementation, albeit to lower capacity than the WT counterpart. Addition of cross-fed amino acids rescued growth of the engineered strains to levels equivalent to the WT counterparts. This is in accordance with previously described consortium characterization (Fig. 1), which shows that not all overproduction levels were sufficient to fully rescue growth of all species.

An important ecological measure of consortium behavior is its population evenness in different environments, which is frequently computed using Pielou evenness index. This measure indicates the extent to which abundances of different species in an ecosystem are even or balanced; a value of 1.0 indicates equal abundances of all species, whereas lower values indicate an ecosystem dominated by a subset of the total species present, and a value of 0 indicates that one species completely dominates the community.

The highest population evenness occurred in the engineered consortia without amino acid supplementation, a setup in which cross-feeding via engineered overproduction and auxotrophies was expected to be maximized. The WT consortium also exhibited a similar trend, showing higher population evenness when amino acid supplementation was omitted. However, the population evenness attained by the WT consortium under this condition was still considerably less than that of the engineered consortium under the same condition. Overall, our results suggest that introduction of cross-feeding can increase population evenness under conditions favoring these beneficial interactions.

### Engineered consortia show higher population evenness than their WT counterpart in the mouse gut.

**FIG 4:**
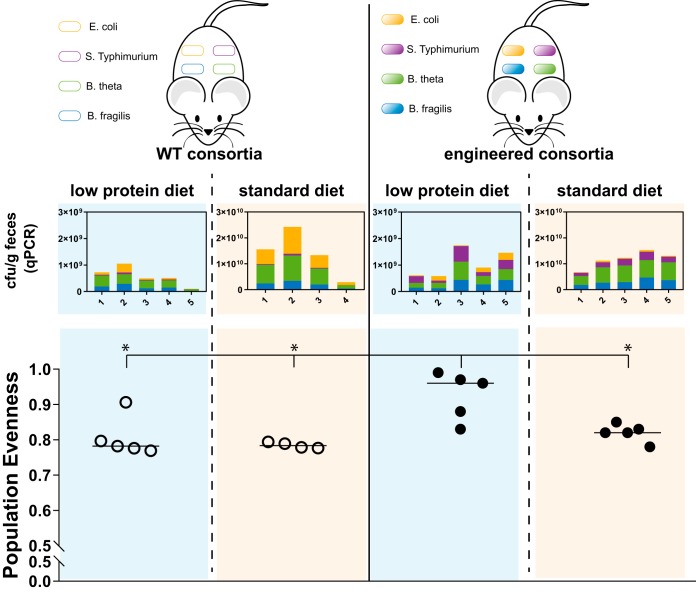
Consortium engineering increases population evenness in the mammalian gut in a diet-dependent manner. Four groups of germfree mice (*n* = 5, except second group from the left, which is *n* = 4) were fed either a low-protein diet or standard diet and inoculated with either the engineered or WT bacterial consortium. Fecal samples 10 days postinoculation were analyzed via strain-specific qPCR to assess concentrations of each consortium species. Population evenness of consortia was calculated. Bar indicates median. The Mann-Whitney test showed significantly increased population of the engineered consortia in mice that were fed the low-protein diet compared to the consortia in the three other groups (*P* values, 0.024, 0.032, and 0.015). Yellow, E. coli; purple, *S.* Typhimurium; green, B. thetaiotaomicron; blue, B. fragilis.

We investigated the behavior of our consortium in the mammalian gut, using gnotobiotic mice as a controlled yet relatively complex environment for evaluation. To investigate the role of amino acid cross-feeding *in vivo*, we altered amino acid levels in the gut by changing the animal’s diet (37). Four groups of five germfree mice were fed standard or low-protein (3%) chow and gavaged with either the WT or engineered consortium (Fig. 4). The consortia were allowed to colonize for 10 days, and then stool samples were collected and interrogated via qPCR with species-specific primers.

We observed consistently higher abundances of all strains in both engineered and WT consortia in mice that were fed with the standard diet. For the engineered consortium, species abundances were higher by a factor of approximately 3 for E. coli, 8 for *S.* Typhimurium, 16 for B. thetaiotaomicron, and 11 for B. fragilis in mice fed a standard versus a low-protein diet. In the case of the WT consortium, *S.* Typhimurium, B. thetaiotaomicron, and B. fragilis concentrations were similarly higher on standard chow (fold changes of approximately 10, 22, and 13, respectively), and WT E. coli concentrations were considerably higher (∼50-fold higher).

Although overall strain abundance was higher in mice that were fed with standard chow, the engineered consortium in mice that were fed with a low-protein diet showed significantly greater population evenness (Mann-Whitney test; *P* values, 0.02, 0.03, and 0.02). These findings are consistent with our *in vitro* results showing the greatest population evenness in the engineered consortium under conditions with low concentrations of the cross-fed amino acids. We also observed a trend toward greater population evenness in the engineered consortium in mice fed standard chow compared to the WT consortium in mice regardless of diet, although this trend was not statistically significant. Interestingly, in mice that were fed with a low-protein diet, *S.* Typhimurium grew about 8-fold better in the engineered consortium than the WT consortium. This finding is consistent with our *in vitro* results, which indicated that engineered *S.* Typhimurium benefits most from the consortium cross-feeding effects. We observed the same trend in mice that were fed with standard diet, albeit to a lesser extent.

## DISCUSSION

We engineered interspecies amino acid cross-feeding in a bacterial consortium and demonstrated that this increased population evenness in environments of various complexity. The ability of an ecosystem to maintain evenness, an important component of species diversity, in the face of different environments or perturbations to the same environment is a component critical to its robustness. We showed that preexisting antagonistic interactions in a bacterial consortium can be overcome by engineered beneficial interactions (Fig. 2). The engineered consortium exhibited increased population evenness *in vitro*, particularly under nutrient-sparse conditions when cross-feeding was favored (Fig. 3).

We found that the engineered consortium also showed increased population evenness in the gnotobiotic mouse gut (Fig. 4). Gnotobiotic mice provide a tractable model for the mammalian gut environment, maintaining the spatial organization and metabolic composition of the host. However, a limitation is that gnotobiotic mice lack the myriad of bacterial species present in a naturally occurring commensal microbiota (38). Despite this limitation, our results provide evidence that engineered bacterial cross-feeding behavior can function in a complex environment and maintain increased population evenness. Notably, while the engineered consortium attained the highest population evenness *in vitro* under conditions lacking the cross-fed amino acids, this was at the expense of overall biomass. However, in the gnotobiotic mouse gut, the engineered consortium attained about the same biomass as its WT counterpart in mice receiving the same diet. Given that the strains in the consortium all originated from the mammalian gut, these findings reinforce the fact that *in vitro* conditions may not mimic important aspects of the host environment, such as availability of complex nutrients and chemical environments in the gut as well as differing spatial distribution of bacterial species, i.e., in large versus small bowel or luminal versus mucosal surfaces. An interesting direction for future work would be to better characterize the niches of the consortium members in the gut, such as through *in situ* localization assays, and determine whether engineering alters spatial localization and other niche behavior.

Engineering diverse, multispecies consortia presents interesting challenges while holding promise for substantial improvements for applications in human or animal health and bioproduction. Bacteria from naturally occurring ecosystems will have preexisting interactions, which are often competitive (4–6). In a given bacterial consortium that is dominated by antagonistic interactions, synthetically introduced positive interactions need to overcome these antagonistic interactions in order to cause a net beneficial effect. We found that in our consortium there was considerable bias toward naturally occurring competitive interactions. Thus, any engineering of these organisms had to overcome this inherent negative bias. We engineered bacteria to overproduce metabolites at different concentrations from low (*S.* Typhimurium, His) to high (B. fragilis, Arg) (Fig. 1). This enabled us to assess the effects of various levels of overproduction in a single consortium. B. fragilis overproduction achieved the most obvious beneficial interactions, and its knockdown *in vitro* had the most profound effect on consortium growth (Fig. 2). Overproduction by the other strains led to reduction of preexisting antagonistic interactions but not to the extent of producing fully beneficial interactions.

Many studies on engineered cross-feeding in microbial consortia have focused on knockouts and complementary metabolism but not overproduction (18, 21). Our findings suggest that relatively high overproduction rates are required in a consortium where multiple members cross-feed from a single or small number of overproducers. We did not observe reduced growth of the overproducing strains, with the exception of B. thetaiotaomicron, which showed a 3-fold reduction in growth (see Fig. S5 in the supplemental material). Our findings have implications for naturally occurring systems and merit further study to understand the extent to which robust cross-feeding has evolved to depend on multiple producers versus high-output single producers.

10.1128/mSystems.00352-19.5 FIG S5 Growth of WT and engineered strains after 24 h, at which point supernatant was collected for cross-feeding experiment (Fig. 1). Overproduction does not affect growth, with the exception of B. thetaiotaomicron (3-fold reduction). Shown are three biological replicates with median indicated as horizontal line. Download FIG S5, EPS file, 0.4 MB. Copyright © 2019 Ziesack et al. 2019 Ziesack et al. This content is distributed under the terms of the Creative Commons Attribution 4.0 International license.

10.1128/mSystems.00352-19.6 FIG S6 Metabolite overproduction and WT and engineered strains. Amino acid production was measured using LC-MS. The average from three biological replicates is shown. Download FIG S6, EPS file, 0.4 MB. Copyright © 2019 Ziesack et al. 2019 Ziesack et al. This content is distributed under the terms of the Creative Commons Attribution 4.0 International license.

We observe that our engineered consortium exhibits increased evenness at the cost of reduced fitness; the engineered consortium grows at ∼10-fold-lower concentration than the WT counterpart (Fig. 2). This is likely in part due to the fact that some of the bacteria overproduce less than is required by the other members (Fig. 1). This imbalance may also make the consortium susceptible to cheaters; in fact E. coli and *S.* Typhimurium could be viewed as “moochers” that benefit most from *Bacteroides* production but do not contribute much themselves. Future work could seek to optimize the balance of overproduction and consumption rates of the consortium members to improve overall consortium fitness while preserving population evenness, using approaches such as rationally engineering overproduction pathways and amino acid uptake transporters in combination with directed evolution via turbidostat.

Our finding that metabolite cross-feeding can lead to increased population evenness, which is an important aspect of species diversity, may have implications for understanding naturally occurring microbial ecosystems as well as engineered communities for biotechnology applications. Indeed, prior work has shown that in a methanotrophic bacterial consortium, amino acid cross-feeding helps to maintain species diversity (15). Species diversity is clearly important in the robustness of microbial ecosystems; an ecosystem with greater genetic diversity as a whole has the potential to better survive in changing environments (e.g., levels of cross-feed nutrients for our consortium). The gut microbiota constitutes a complex host-microbial ecosystem, and maintaining species diversity in this context has important implications for human health. Our internal microbial ecosystems undergo a variety of alterations due to factors including normal physiologic changes such as exercise or pregnancy, pathological states due to disease, modifications to our diets, and medical interventions. Thus, mechanisms such as cross-feeding that promote maintenance of species diversity in the human microbiome in the face of perturbing factors may be critical to maintaining host function. From the perspective of an ecosystem, there is a long-term evolutionary fitness advantage to maintaining species diversity, which may be supported by naturally occurring metabolite cross-feeding mechanisms such as those that we introduce artificially here.

## MATERIALS AND METHODS

### Auxotroph engineering.

For auxotroph generation in the E. coli NGF-1 strain, we introduced multiple knockouts using sequential P1 transduction (39) from the Keio knockout collection (40). Flipout of kanamycin cassettes was done using pCP20 (41). In brief, for P1 transduction we prepared phage by diluting an overnight culture of the donor strain 1:100 in LB with 0.2% glucose, 5 mM CaCl_2_, and 25 mM MgCl_2_ and incubated it for 1 to 2 h at 37°C until slightly turbid. We then added 40 μl P1 lysate and continued growth for 1 to 3 h at 37°C while shaking the culture until lysed. Lysate was then filtered with a 20-μm sterile filter and stored in the refrigerator. For transduction, we harvested a 2-ml overnight culture of the recipient strain and resuspended it in 2 ml LB with 5 mM CaCl_2_ and 100 mM MgSO_4_. We then mixed 100 μl donor lysate with 100 μl recipient, incubated the mixture for 30 min at 37°C, added 200 μl sodium citrate (1 M, pH 5.5) and 1 ml LB, and incubated the mixture for another 1 h at 37°C. Cells were harvested, resuspended in 100 μl LB with 100 mM sodium citrate, and plated on LB-kanamycin (Kan) plates (75 μg/ml). The transduced kanamycin cassette was then removed using pCP20 according to protocol. We transformed pCP20 via electroporation, and transformants were selected on LB agar plates supplemented with 100 μg/ml carbenicillin and grown at 30°C. Single colonies were restreaked on LB without drugs and incubated for 10 h at 42°C. From there, single colonies were restreaked on LB plates without drugs and grown overnight at 37°C. Colonies were checked for carbenicillin and kanamycin sensitivity and further confirmed via PCR at respective loci. This procedure was repeated until all knockouts were introduced.

Engineering of *S.* Typhimurium LT2 required generation of single-knockout strains that contained pKD46 integrated into the genome, which allowed for linear DNA integration using lambda red recombination (41). We then introduced the knockouts into the *S.* Typhimurium strain through sequential P22 transduction and pCP20 flipout analogous to E. coli engineering. Single-knockout strains were generated by PCR amplifying a kanamycin resistance cassette from pKD13, generating linear fragments that contained upstream and downstream homology to the gene of interest and the kanamycin cassette with FLP recombination target (FRT) sequences. Fragments were introduced via electroporation and selected on LB agar plates supplemented with 50 μg/ml kanamycin. Sequential P22 transduction and pCP20 flipout were essentially performed as described above for P1 transduction, but lysis was done overnight.

For knockout generation for both B. thetaiotaomicron and B. fragilis, we used pExchange KO vectors as described previously (42). Briefly, we introduced 750-bp flanking regions for genes of interest adjacent to each other into the vector. The vector contains erythromycin resistance as a positive marker and thymidine kinase as a counterselection marker. Cloning was done in *pir*^+^ E. coli strains, and vectors were transferred to MFDpir for conjugation (43). Conjugation was done according to protocol with minor changes. In brief, 5 drops of overnight culture of E. coli donor was inoculated in LB supplemented with 300 μM diaminopimelic acid (DAP), and 5 drops of recipient overnight culture was inoculated in 50 ml basal medium. Both cultures were grown for about 2 h (E. coli aerobically, *Bacteroides* spp. anaerobically) until E. coli culture was highly turbid and *Bacteroides* culture was just slightly turbid. Subsequently, 9 ml recipient and 3 ml donor were combined and spun down for 10 min at 4,000 rpm together. The pellet was resuspended in 100 μl fresh basal medium with 300 μM DAP and pipetted on basal medium agar plates without cysteine and supplemented with 300 μM DAP. The cells were incubated at 37°C aerobically face up for up to 20 h, scraped off, and resuspended in 10% glycerol. Dilutions were plated on basal agar plates supplemented with 10 μg/ml erythromycin and incubated at 37°C anaerobically for 2 to 3 days. Single colonies were restreaked in the presence of erythromycin and grown for another 2 days. Ten single colonies were inoculated in basal medium without drug and grown overnight. Five hundred microliters of each culture was mixed, spun down, and resuspended in 10% glycerol. We then plated different dilutions on basal medium plates supplemented with 5-fluoro-2-deoxyuridine (FuDR) (200 μg/ml) and incubated them at 37°C anaerobically for 3 days. Knockouts were verified via PCR. This procedure was repeated multiple times to obtain the multiple auxotroph strains.

### Overproducer selection.

Overproducers were generated by selecting for mutants that could grow on minimal medium agar plates supplemented with antimetabolites [E. coli, 5 mg/ml norleucine for Met overproduction; *S.* Typhimurium, >0.7 mg/ml beta-(2-thiazolyl)-dl-alanine for His overproduction; B. thetaiotaomicron, 50 μg/ml 4-methyltryptophan for Trp overproduction; B. fragilis, 80 μg/ml canavanine for Arg overproduction]. Single colonies that showed halos were restreaked, and overproduction was measured using a bioassay. In brief, for screening of overproducing mutants the isolated strains were grown overnight at 37°C with shaking aerobically (for E. coli and *S.* Typhimurium) or anaerobically without agitation (for *Bacteroides* spp.). Supernatant was harvested and diluted 1:1 with fresh medium, E. coli auxotrophs were inoculated, and their growth was recorded after 24 h. For E. coli NGF-1 overproducers, we used an *S.* Typhimurium auxotroph instead, since its colicin production prevented the E. coli biosensor from growing. Confirmed overproducers were further quantified using LC-MS.

### LC-MS for overproduction measurements.

To quantitate amino acid levels in overproducer supernatants, a standard curve was obtained using freshly prepared amino acid standards dissolved in growth medium (1 mM, 500 μM, 100 μM, 50 μM, 10 μM l-methionine, l-histidine, l-tryptophan, and l-arginine each). To prepare for high-performance liquid chromatography-mass spectrometry (HPLC-MS) analysis, 0.5 ml sample or standard was added to 1.5 ml ice-cold methanol and incubated on ice for 10 min. The mixture was centrifuged for 5 min at 15,000 rpm, and 500 μl supernatant was vacuum concentrated and resuspended in 50 μl methanol. Samples were kept on ice or at 4°C. HPLC-MS analysis of standards and extracts was carried out using an Agilent 1260 Infinity HPLC system equipped with an Agilent Eclipse Plus C_18_ column (100 by 4.6 mm; particle size, 3.5 mm; flow rate, 0.3 ml/min; solvent A, double-distilled water [ddH_2_O]-0.1% [vol/vol] formic acid; solvent B, acetonitrile; injection volume, 4 ml) connected to an Agilent 6530 accurate-mass quadrupole time of flight (Q-TOF) instrument. The following gradient was used (time/minute, percent B): 0, 0; 0.5, 0; 14, 100; 19, 100; 20, 0, 25, 0. The mass spectrometer was operated in positive mode, and the autosampler was kept at 4°C. After HPLC-MS analysis, extracted ion current (EIC) peaks were automatically integrated using the MassHunter Workstation software (version B.07.00). A plot of peak area versus amino acid concentration was used to generate a linear fit.

### Sequencing.

Bacterial cultures were prepared in rich medium (basal for *Bacteroides* spp. and LB for E. coli and *S.* Typhimurium). Genomic DNA (gDNA) extraction was performed using the Wizard genomic DNA purification kit (Promega) according to protocol. The extracted gDNA was sheared using Covaris DNA shearing, and the library was prepared using Kapa Biosystems DNA Hyper Prep NGS library (Dana-Farber Core MBCFL Genomics). Sequencing was performed on the Illumina MiSeq instrument, with the 150-bp paired-end (PE150) reagents; raw reads can be found under the accession number PRJNA557416. Sequences were analyzed for SNPs using Geneious software and published genome sequences (E. coli, CP016007.1; *S.* Typhimurium, NC_003197; B. thetaiotaomicron, AE015928; B. fragilis, NC_016776).

### Growth and medium conditions.

All basal media (20 g/liter proteose peptone, 5 g/liter yeast extract, 5 g/liter sodium chloride, 1 g/liter l-cysteine, 0.5% glucose, 5 mg/ml potassium phosphate, 5 mg/liter hemin) and coculture media (M9 salts [0.2 g/liter Na_2_HPO_4_, 90 mg/liter KH_2_PO_4_, 30 mg/liter NH_4_Cl, 15 mg/liter NaCl]; 1 mM MgSO_4_; 10 μg/ml heme; 0.1 mM CaCl_2_; 1 μg/ml niacinamide, vitamin B_12_, and thiamine; 400 μg/ml l-cysteine; 0.3% bicarbonate buffer; 2.5 ng/ml vitamin K; 2 μg/ml FeSO_4_·7H_2_O; and carbon sources and amino acid supplementation as described in Results) were preincubated for at least 24 h anaerobically before use. In initial experiments, we used 0.5% starch, which we later optimized to 0.2% (resulting in lower viscosity); the observed growth differences were not statistically significant between those two conditions (see Fig. S7 in the supplemental material). *Bacteroides* spp. were inoculated from glycerol stock into basal medium and grown overnight, and 400 μl was inoculated in 5 ml basal medium and grown for 2 h anaerobically. Cells were spun down, washed twice in phosphate-buffered saline (PBS), and diluted in growth medium as described for each experiment in Results. E. coli and *S.* Typhimurium were inoculated from glycerol stock into LB and grown overnight at 37°C while shaking. One hundred microliters of culture was then inoculated into preincubated LB, grown anaerobically for 2 h, diluted, washed in PBS, and diluted into coculture medium as described above.

10.1128/mSystems.00352-19.7 FIG S7 Growth after 24 h in medium supplemented with 0.5% or 0.2% starch. Growth did not differ significantly for any of the strains (Mann-Whitney test, *P* values > 0.1). Download FIG S7, EPS file, 0.5 MB. Copyright © 2019 Ziesack et al. 2019 Ziesack et al. This content is distributed under the terms of the Creative Commons Attribution 4.0 International license.

### Multiplex qPCR.

We designed strain-specific primer/probe-fluorophore pairs according to IDT protocol (see Table S1 in the supplemental material). We chose strain-specific genes by multiple genome alignment between the strain of interest, the other consortium members, and closely related strains using Mauve (44). Multiplex qPCR was used to quantify each strain in coculture by using a standard curve obtained by plating late-log-phase cultures grown in rich medium. In brief, each strain was grown from overnight culture for ∼5 h until reaching an optical density (OD) of about 1. Cells were then counted by plating. Cultures were mixed, diluted, and frozen at −80°C for use as a standard curve. Samples were diluted 1:10 in ddH_2_O, snap-frozen in liquid nitrogen, and stored at least overnight at −80°C. Growth curve and sample were thawed together and prepared in a 5-μl Primetime Mastermix (IDT) with 1 μl primer-probe mixture (final concentrations, 100 nM for primers and 50 nM for probes). The qPCR was run with the following program: 20 min at 98°C (to boil the cells and denature gDNA), followed by 40 cycles of 60°C and 98°C.

10.1128/mSystems.00352-19.8 TABLE S1 qPCR probes and primers. Download Table S1, EPS file, 0.5 MB. Copyright © 2019 Ziesack et al. 2019 Ziesack et al. This content is distributed under the terms of the Creative Commons Attribution 4.0 International license.

### Statistical inference of interactions within consortia.

We adapted our previously published statistical method for inferring a dynamical systems model from microbial time-series data (33, 34). Briefly, our model assumes that the rate of change over time of each microbe in the ecosystem is related to its own abundance as well as abundances of the other microbes in the ecosystem. Our model is fully Bayesian and infers the posterior probability distribution over the qualitative network of microbe-microbe interactions (e.g., probability of an edge being present in the network) as well as the quantitative strengths of the interactions present. As we have previously described (33), the qualitative network can be interpreted in terms of Bayes factors (BFs), a standard Bayesian alternative for hypothesis testing that quantifies the evidence for one model over another. For our method, the BF on network edges quantifies the evidence for existence of the interaction versus absence of the interaction. A standard interpretation of BFs is BF between 3 and 10 equals substantial evidence and BF of >10 equals strong evidence.

To be precise, our model is based on discrete time stochastic generalized Lotka Volterra (gLV) dynamics:

$$x_{k+1,i}=x_{k,i}+x_{k,i}\left(r_{i}+\overset{4}{\underset{j=1}{\sum }}a_{i,j}z_{i,j}x_{k,j}\right)\Delta_{k}+\sqrt{\Delta_{k}}\left(w_{k+1,i}-w_{k,i}\right)$$

where ***x***_*k,i*_ is the abundance of species *i* at time point *k*; ***r****_i_* is the growth rate of species *i*; ***a***_*i,j*_ is the interaction coefficient and ***z***_*i,j*_ is a binary variable indicating the presence or absence of an edge (interaction) between species *j* and *i*; Δ*_k_* is the time difference between time points *k* + 1 and *k*; and ***w****_k,i_* is a Brownian motion term. The Brownian motion has variance $v_{i}^{w}$. We place prior probability distributions on the variables defined above: ***z***_*i,j*_ ∼ Bernoulli(0.5); ***r****_i_*|***v***^***r***^ ∼ Normal(0,***v***^***r***^); ***a***_*i,j*_|***v***^***a***^ ∼ Normal(0,***v***^***a***^).

Note that our prior probability distribution for the indicator variable, ***z****_i_*_,_*_j_*, for presence or absence of interactions, expresses maximum uncertainty, e.g., no *a priori* assumption about the presence or absence of an interaction. We place the following prior probability distributions on the variance parameters in our model: ***v***^***r***^ ∼ Inv − χ^2^(η_***r***_, θ_***r***_); ***v***^***a***^ ∼ Inv − χ^2^(η_***a***_, θ_***a***_); $v_{i}^{w}\;∼\text{ Inv}-\chi^{2}\left(\eta_{w},\;\theta_{w}\right)$.

In our model, η***_r_***, η***_a_***, η***_w_*** = 0.1 with θ***_r_*** = 0.01, θ***_a_*** = 10^−14^, and θ***_w_*** = 10^4^, specifying relatively diffuse (uninformative) priors. We perform inference using a simplified version of our previously described Markov chain Monte Carlo (MCMC) algorithm that omits clustering (34), with 5,000 iterations (1,000 burn-in iterations.)

The gLV model described above allows for only pairwise interactions, so we also tested an extended model with higher-order interactions. Specifically, we included 3rd-order or 4th-order interactions, i.e.,

$$+z_{i}^{\left(3\right)}a_{i}^{\left(3\right)}\underset{j\neq i}{\prod }x_{k,j}\text{ or}+z_{i}^{\left(4\right)}a_{i}^{\left(4\right)}\overset{4}{\underset{j=1}{\prod }}x_{k,j}$$

appearing on the right-hand side of the gLV dynamics equation above. The superscripts “(3)” and “(4)” denote higher-order indicator variables $z_{i}^{\left(\cdot \right)}$ and interaction coefficients $a_{i}^{\left(\cdot \right)}$ for orders 3 and 4, respectively. However, when we applied the model to our data, pairwise interactions dominated, and the indicator variables associated with the higher-order interactions had Bayes factors near zero. From these analyses, we can conclude that the presence of higher-order interactions was not supported by our data. However, it is possible that such interactions actually exist in the ecosystem but that our data are insufficient for rigorous inference using our statistical method.

### Calculation of population evenness (Pielou’s evenness).

Species diversity (Pielou’s evenness) was calculated according to the given formula:

$$\text{Pielou’s evenness index}=\frac{-\sum_{i=1}^{4}p_{i}\cdot \text{ln}(p_{i})}{\text{ln}(S)}$$

where *p_i_* refers to the population ratio of a given strain in the consortium of four strains. *S* is the number of species.

### *In vivo* experiments.

Adult (6 to 8 weeks) male Swiss Webster germfree mice bred in-house at the Massachusetts Host-Microbiome Center were used. Animals were fed either on standard chow in the facility for the entire experiment or on low-protein diet (3% custom diet [Envigo], doubly irradiated) beginning 10 days prior to the experiment and continuing for its duration. To prepare bacteria for gavage, we grew each strain to mid-log phase, plated it for counting, and snap-froze aliquots. For gavage, aliquots were thawed, spun down, combined to achieve concentrations of approximately 10^7^ per bacteria per gavage, and resuspended in 200 μl 1× PBS with 0.05% l-cysteine for gavage. After gavage, mice were transferred to Optimice cages and maintained gnotobiotic for 10 days. Fecal samples were collected before gavage and at 10 days and snap-frozen for storage at −80°C.

### Molecular analysis of *in vivo* samples.

DNA was extracted from fecal samples using the Zymobiomic 96 DNA kit with the following modification: we omitted the Silicon-A-HRC plate wash. Cells were lysed in a bead beater at speed 20 for 10 min, and plates were turned and lysed for another 10 min at the same speed. We added an additional 3-min incubation step for binding buffer and additional 5-min incubation steps when transferring to Zymo-Spin-I-96-Z plates. Elution was done in 50 μl Zymobiomic DNase/RNase-free water.

Direct multiplex probe-based qPCR was done on extracted DNA samples as described above. For standard curves, we used plated overnight cultures spiked into germfree fecal samples and extracted them as described above.
